# Supplementary material for: A New Isolated Fungus and Its Pathogenicity for Apis mellifera Brood in China
Source: Microorganisms. 2024 Feb 1;12(2):313. doi: 10.3390/microorganisms12020313 (PMC10892447; doi:10.3390/microorganisms12020313)
Supplement: Supplementary file 1 [file microorganisms-12-00313-s001.zip › microorganisms-2709044-supplementary.pdf]

Tables

Supplemental Table S1. Pathogenicity of different concentrations of R. oryzae spores to honeybee larvae

	Total number of larvae	Death number	Mortality	Mean+SD	Corrected Mortality	LT-P Probit model	LT50 (hours)	Lower and upper 95% Fiducial Limits (hours)
T0	72	11.5	15.97	13.57±3.21C	----	----	----	----
	72	8.66	12.02		----			
	72	9.17	12.73		----			
T1	65	12.33	17.84	17.39±2.69C	4.94	----	----	----
	65	11.33	17.43		4.47			
	68	11.50	16.91		3.86			
T2	68	16.66	24.50	21.76±5.88BC	12.65	----	----	----
	67	17.83	21.61		9.30			
	66	12.66	19.18		6.49			
T3	70	24.16	34.51	35.88±2.70B	24.23	Y=-3.654+1.635x	171.85	106.98
	72	25.85	35.88		25.81			1319.95
	72	26.83	37.26		21.41			
T4	69	57.5	83.30	84.94±2.16A	80.68	Y=-8.310+5.816x	26.80	22.33
	70	60.16	85.94		83.73			30.80
	94	80.33	85.46		83.18			
T5	72	62.33	86.57	85.51±2.41A	84.46	Y=7.970+5.595x	25.50	20.99
	72	61.00	84.72		82.32			30.03
	70	59.67	85.23		82.91			

Note: Mortality=number of total dead larvae/total number of larvae in each replication*100. Corrected mortality= ((number of total dead larvae from treatment group-number of total dead larvae from control group)/ (100- number of total dead larvae from control group)) *100. Treatments followed by different letters indicate significant differences at $p<0.05$. T1, T2, T3, T4, T5 stands for *R. oryzae* spore inoculated larva in 1×10^2, 1×10^3, 1×10^4, 1×10^5, and 1×10^6 respectively, T0 is control group, in which larvae were fed on normal diet. The experiments were conducted in triplicates. Probit (p) = Intercept+BX (Covariates X were transformed using the base 10.00 logarithm).

Supplemental Table S2 Death rate of honeybee larvae inoculated with different concentrations of *R. oryzae* spores, over incubation time.

Concentration		24h	48h	72h	96h	144h
0	Mortality	13.89	13.89	15.27	16.67	18.05
	(%)	9.72	9.72	11.11	11.11	16.67
		6.9	8.33	11.11	16.67	16.67
	Mean±SD	10.17±3.52	10.65±2.89	12.50±2.4	14.81±3.21	17.13±0.80
$1x10^2$	Mortality	13.85	15.38	15.38	18.46	26.15
	(%)	6.15	10.77	14.71	21.54	29.23
		11.76	13.23	16.92	16.18	23.53
	Mean±SD	10.59±3.98	13.13±2.30	15.67±1.13	18.72±2.69	26.30±2.85
$1x10^3$	Mortality	11.76	14.71	19.12	22.06	45.59
	(%)	14.93	17.91	22.39	26.87	40.30
		12.12	15.15	15.15	15.18	30.30
	Mean±SD	12.93±1.74	15.92±1.73	18.87±3.63	21.37±5.88	38.73±7.76
$1x10^4$	Mortality	21.43	21.42	25.71	32.86	55.71
	(%)	25	25	25	27.78	61.11
		27.78	30.55	31.94	31.94	55.55
	Mean±SD	24.74±3.18	25.65±4.6	27.55±3.81	30.86±2.70	57.45±3.16
$1x10^5$	Mortality	52.17	69.56	79.79	98.55	100
	(%)	51.43	74.62	90	100	100
		54.26	71	91.48	95.74	100
	Mean±SD	52.62±1.47	71.96±5.40	91.60±2.35	98.61±2.40	100±0.00
$1x10^6$	Mortality	52.78	77.78	93.05	95.83	100
	(%)	48.61	70.83	88.89	100	100
		51.43	67.14	92.85	100	100
	Mean±SD	50.94±2.13	71.91±5.40	91.60±2.35	98.61±2.41	100±0.00

Supplemental Table S3 Death number of honeybee larvae inoculated with *R. oryzae* spores with different concentration over incubation time.

Treatment	Total number of larvae	Death number of larvae				
		24h	48h	72h	96h	144h
T0	72	10	10	11	12	13

	72	7	7	8	8	12
	72	5	6	8	12	12
T1	65	9	10	11	12	17
	65	4	7	10	14	19
	68	8	9	10	11	16
T2	68	8	10	13	15	31
	67	10	12	15	18	27
	66	8	10	10	12	20
T3	70	15	15	18	23	39
	72	18	18	18	20	44
	72	20	22	23	23	40
T4	69	36	48	55	68	69
	70	36	52	63	70	70
	94	51	67	86	90	94
T5	72	38	56	67	69	72
	72	35	51	64	72	72
	70	36	47	65	70	70

Supplemental Table S4 Multiple comparisons of virulence of different concentration *R. oryzae* spores to honeybee larvae

(I) Concentration	(J) Concentration	Mean Difference(I-J)	Sig.	95% Fiducial Limits	
				Lower	Upper
0 (ck)	$1x10^2$	-3.91	.242	-10.83	3.01
	$1x10^3$	-6.54	.062	-13.46	.375
	$1x10^4$	-12.23*	.002	-19.15	-5.32
	$1x10^5$	-83.28*	.000	-90.20	-76.36
	$1x10^6$	-83.79*	.000	-90.71	-76.87
$1x10^2$	0	3.91	.242	-3.01	10.83
	$1x10^3$	-2.63	.423	-9.55	4.28
	$1x10^4$	-8.32*	.022	-15.24	-1.40
	$1x10^5$	-79.37*	.000	-86.29	-72.45
	$1x10^6$	-79.88*	.000	-86.80	-72.96
$1x10^3$	0	6.54	.062	-.37	13.46
	$1x10^2$	2.63	.423	-4.28	9.55
	$1x10^4$	-5.69	.098	-12.60	1.23
	$1x10^5$	-76.74*	.000	-83.65	-69.81
	$1x10^6$	-77.25*	.000	-84.16	-70.33
$1x10^4$	0	12.23*	.002	5.31	19.15
	$1x10^2$	8.32*	.022	1.40	15.24
	$1x10^3$	5.69	.098	-1.23	12.61
	$1x10^5$	-71.05*	.000	-77.96	-64.64
	$1x10^6$	-71.56*	.000	-78.47	-64.64
$1x10^5$	0	83.28*	.000	76.36	90.20
	$1x10^2$	79.37*	.000	72.45	86.29
	$1x10^3$	76.73*	.000	69.81	83.65
	$1x10^4$	71.04*	.000	64.13	77.96
	$1x10^6$	-.51	.087	-7.43	6.41
$1x10^6$	0	83.79*	.000	76.87	90.71
	$1x10^2$	79.88*	.000	72.96	86.80
	$1x10^3$	77.25*	.000	70.33	86.80
	$1x10^4$	71.56*	.000	64.64	78.47
	$1x10^5$	.51	.000	-6.41	7.43

Note: * indicate significant differences at $p<0.05$

Supplemental Table S5 Multiple comparisons of virulence of *R. oryzae* spores to honeybee larvae over time

(I) Time	(J) Time	Mean Difference(I-J)	Sig.	95% Fiducial Limits	
				Lower	Upper
24	48	-7.82*	.000	-9.15	-6.48
	72	-15.21*	.000	-16.90	-13.53
	96	-19.61*	.000	-21.76	-17.47
	144	-29.61*	.000	-32.05	-27.16
48	24	7.82*	.000	6.48	9.15
	72	-7.40*	.000	-9.17	-5.63
	96	-11.80*	.000	-13.99	-9.61
	144	-21.80*	.000	-24.43	-19.16
72	24	15.21*	.000	13.53	16.90
	48	7.40*	.000	5.63	9.17
	96	-4.40*	.000	-6.50	-2.30
	144	-14.39*	.000	-16.79	-12.00
96	24	19.61*	.000	17.47	21.76
	48	11.80*	.000	9.61	13.99
	72	4.40*	.000	2.30	6.50
	144	-9.99*	.000	-12.01	-7.97
144	24	29.61*	.000	27.16	32.05
	48	21.80*	.000	19.16	24.43
	72	14.39*	.000	12.00	16.79
	96	9.99*	.000	7.97	12.01

Note: * indicate significant differences at $p<0.05$

Supplemental Table S6 Multivariate Tests

Multivariate Tests

Effect		Value	F	Hypothesis df	Error df	Sig.
time	Pillai’s Trace	.986	164.048[b]	4.000	9.000	.000
	Wilks’Lambda	.014	164.048[b]	4.000	9.000	.000
	Hotelling’s Trace	72.910	164.048[b]	4.000	9.000	.000
	Roy’s Largest Root	72.910	164.048[b]	4.000	9.000	.000
Time*Concentration	Pillai’s Trace	2.045	2.512	20.000	48.000	.005
	Wilks’Lambda	.002	8.375	20.000	30.799	.000
	Hotelling’s Trace	52.542	19.703	20.000	30.000	.000
	Roy’s Largest Root	44.698	107.276[c]	5.000	12.000	.000
